# Mutations in the *SdhB* and *SdhD* Genes Confer Resistance to Succinate Dehydrogenase Inhibitor Fungicides in Australian Populations of *Leptosphaeria maculans*

**DOI:** 10.1111/mpp.70364

**Published:** 2026-11-06

**Authors:** Ko‐Hsuan Tsao, Alexander Idnurm, Angela P. Van de Wouw, Alec J. McCallum

**Affiliations:** ^1^ School of BioSciences The University of Melbourne Melbourne Victoria Australia

**Keywords:** blackleg, *Brassica napus*, fungicide resistance, *Leptosphaeria maculans*, SDHI

## Abstract

Fungicide resistance is a threat to the control of fungal diseases of crops and humans. *Leptosphaeria maculans* causes blackleg disease, a major disease of canola worldwide and reliance on fungicide applications to control blackleg has increased. Succinate dehydrogenase inhibitor (SDHI) fungicides are widely used but are at high risk of resistance development. This study reports the first detection of SDHI resistance in *L. maculans* worldwide as part of a decade‐long survey for fungicide resistance across Australia. SDHI resistant isolates were collected and characterised using in vitro and in planta assays. SDHI resistance‐associated mutations were identified and validated through genetic mapping, sequencing and functional complementation. Three single‐nucleotide polymorphisms in *SdhB* and *SdhD* genes, *SdhB*‐H277Y, *SdhB*‐H277L and *SdhD*‐D144G, were confirmed to confer reduced SDHI sensitivity. Molecular markers for all three mutations were developed and validated for resistance monitoring, accurately differentiating resistant and susceptible genotypes in mapping populations. Deep amplicon sequencing revealed that SDHI resistance alleles were present at frequencies below 1% in most populations, although a small number of populations exceeded this level. Collectively, these findings provide the first evidence that SDHI resistance has emerged in *L. maculans* populations and highlight that fungicide efficacy will likely be impacted. The identification of causal mutations and development of monitoring tools provides an important foundation for surveillance and early resistance management for blackleg control.

## Graphical abstract

Resistance to the SDHI fungicides is discovered and characterised in a major oilseed pathogen, *Leptosphaeria maculans*. Mutations are found in genes encoding two subunits of the SDH complex, and their prevalence assayed in populations.

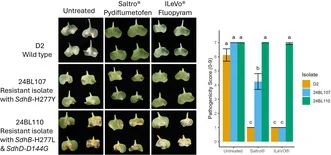

## 1 Introduction

Blackleg disease, caused by the ascomycete *Leptosphaeria maculans*, is one of the most important diseases of canola (oilseed rape, *Brassica napus* ) worldwide, causing 5%–30% yield loss globally (Sprague et al. 2024). Blackleg disease is typically controlled through integrated disease management strategies that incorporate cultural practices, host genetics and fungicides. Cultural practices aim to reduce exposure to inoculum, principally from canola stubble, through crop rotations and stubble management (Sprague et al. 2024). However, cultural practices often become unfeasible due to the intensification of canola production along with changes to farming systems that promote stubble retention (Van de Wouw, Marcroft, et al. 2021). Host genetics include both qualitative and quantitative resistance to blackleg disease and has proven to be the most economically viable strategy for blackleg control (Sprague et al. 2024). However, host genetic resistance is regularly overcome due to the rapid evolution of fungal populations (Sprague et al. 2006; Van de Wouw et al. 2014). As such, farmers have an increasing reliance on fungicides for control of blackleg disease; for example, Australian farmers used on average 2.5 applications per canola growing season by 2023 (McCallum, Idnurm, and Van de Wouw 2025) and UK farmers apply three fungicides per season (Chantry et al. 2024).

In Australia, currently, three groups of fungicides are commercially registered for blackleg control: demethylation inhibitors (DMIs, Fungicide Resistance Action Committee [FRAC] group 3), quinone outside inhibitors (QoIs, FRAC group 11) and succinate dehydrogenase inhibitors (SDHIs, FRAC group 7) (McCallum, Idnurm, and Van de Wouw 2025). DMIs target sterol 14α‐demethylase, encoded by *erg11/CYP51* and involved in ergosterol biosynthesis (Yang et al. 2020). DMIs have been used for blackleg control for over two decades with the first DMI fungicide, Impact (flutriafol, in‐furrow), released in 1997, followed by Jockey (fluquinconazole, seed dressing) in 2003 and Prosaro (tebuconazole and prothioconazole, foliar) in 2011 (Van de Wouw, Scanlan, et al. 2021). QoIs inhibit the respiration of fungi by targeting the quinone outside (Qo) site of cytochrome *b* (Fernández‐Ortuño et al. 2008). In Australia, three QoI products are registered to control blackleg: Veritas Opti (azoxystrobin and tebuconazole [DMI], foliar), Maxentis (azoxystrobin and prothioconazole [DMI], foliar) and Mirador (azoxystrobin, foliar) (McCallum, Idnurm, Sheedy, et al. 2025). SDHIs inhibit respiration and the citric acid cycle by targeting the mitochondrial succinate dehydrogenase (SDH, Complex II), which consists of four subunits: SDHA, SDHB, SDHC and SDHD (Sierotzki and Scalliet 2013). Four SDHI active ingredients are registered for blackleg control: bixafen, pydiflumetofen, fluopyram and fluxapyroxad, as Aviator (bixafen and prothioconazole [DMI], foliar), Saltro (pydiflumetofen, seed dressing), Miravis (pydiflumetofen, foliar), ILeVO (fluopyram, seed dressing) and Revystar (fluxapyroxad and mefentrifluconazole [DMI], foliar).

Increased reliance on fungicides has driven the emergence of fungicide resistance to DMIs in *L. maculans* populations, creating an urgent need to better understand resistance development and persistence in the field. Fungicide resistance refers to a heritable decrease in sensitivity toward fungicides, enabling pathogens to grow and reproduce under the pressure from fungicide applications (Yin et al. 2023). The first reported case of fungicide resistance in *L. maculans* was in Australia in 2015, as discovered using an in planta assay (Van de Wouw et al. 2017). That study found that DMI resistance in isolates was caused by the insertions of DNA into the regulatory region of the *erg11/CYP51* gene, and similar mutations have since been reported throughout Europe (Van de Wouw, Scanlan, et al. 2021; Fajemisin et al. 2022; King et al. 2024; Scanlan et al. 2024). QoI resistance is usually conferred by single‐nucleotide polymorphisms (SNPs) that produce an amino acid substitution, such as G143A (substitution of glycine by alanine at position 143) of cytochrome *b* in several plant fungal pathogens (Fernández‐Ortuño et al. 2008). No QoI resistance has been identified to date in *L. maculans* (McCallum, Idnurm, Sheedy, et al. 2025). SDHI resistance has been reported to be caused by SNPs in genes encoding the SDH subunits with many examples of SDHI resistance in pathogens, such as *Alternaria alternata* , *Botrytis cinerea* and *Fusarium* species (Avenot et al. 2014; Hu et al. 2016; Song et al. 2026). Prior to 2024, no SDHI resistance in *L. maculans* had been reported in Australia despite widespread surveying having been conducted since 2015 (McCallum, Idnurm, and Van de Wouw 2025).

In the current study, we report on the detection of the first SDHI‐resistant *L. maculans* isolates, the genetic basis of resistance and examine the frequency of SDHI resistance in field populations. This study details the collection of SDHI resistance isolates, the characterisation and validation of mutations conferring SDHI resistance in *L. maculans* and resolves the frequencies of *Sdh* mutations in Australian *L. maculans* populations.

## 2 Results

### 2.1 Detection of SDHI Resistance in *L. maculans* in a 2024 Field Fungicide Sensitivity Survey

In 2024, the sensitivity of 110 *L. maculans* populations toward three SDHI commercial fungicides (Table 1) was tested using an in planta assay (Van de Wouw et al. 2017). Stubble from each population was used to inoculate seedlings treated with three registered SDHI fungicides: Saltro (pydiflumetofen), ILeVO (fluopyram) and Aviator XPro (bixafen and prothioconazole). Disease severity on cotyledons was scored, and each population was given a resistance score (RS) based on the level of disease on the fungicide‐treated plants compared to the untreated, which was converted to a rating of ‘none’, ‘low’, ‘moderate’ or ‘high’ (Figure 1). For each of the chemistries, between 80.4% and 98.7% of the populations had a resistance rating of none or low, suggesting no fungicide resistance is present in these populations. The frequency of moderate disease rated populations varied from 1% to 17.5%, and for Saltro (pydiflumetofen) and ILeVO (fluopyram), 2.1% and 1.0% of the populations were categorised as ‘high’, respectively. No populations had a high disease rating toward Aviator XPro (bixafen and prothioconazole). The detection of moderate and high resistance ratings in some populations indicated the potential for the presence of SDHI resistance mutations within these populations.

**TABLE 1:** Details of fungicides used in experiments.

Trade name	Active ingredient/s	FRAC class	FRAC group	Company	Application	Registered in canola	Experiments
Saltro	Pydiflumetofen	SDHI	7	Syngenta	Seed dressing	Yes	Fungicide resistance survey and in planta assay
ILeVO	Fluopyram	SDHI	7	BASF	Seed dressing	Yes	Fungicide resistance survey and in planta assay
Aviator XPro	Bixafen + Prothioconazole	SDHI + DMI	7 + 3	Bayer	Foliar	Yes	Fungicide resistance survey and in planta assay
Miravis	Pydiflumetofen	SDHI	7	Syngenta	Foliar	Yes	In planta assay
Xzemplar	Fluxapyroxad	SDHI	7	BASF	Foliar	No	In planta assay
Revystar	Fluxapyroxad + Mefentrifluconazole	SDHI + DMI	7 + 3	BASF	Foliar	Yes	In planta assay
Proviso	Prothioconazole	DMI	3	Adama	Foliar	Yes	In planta assay
Belanty	Mefentrifluconazole	DMI	3	BASF	Foliar	No	In planta assay

**FIGURE 1:**
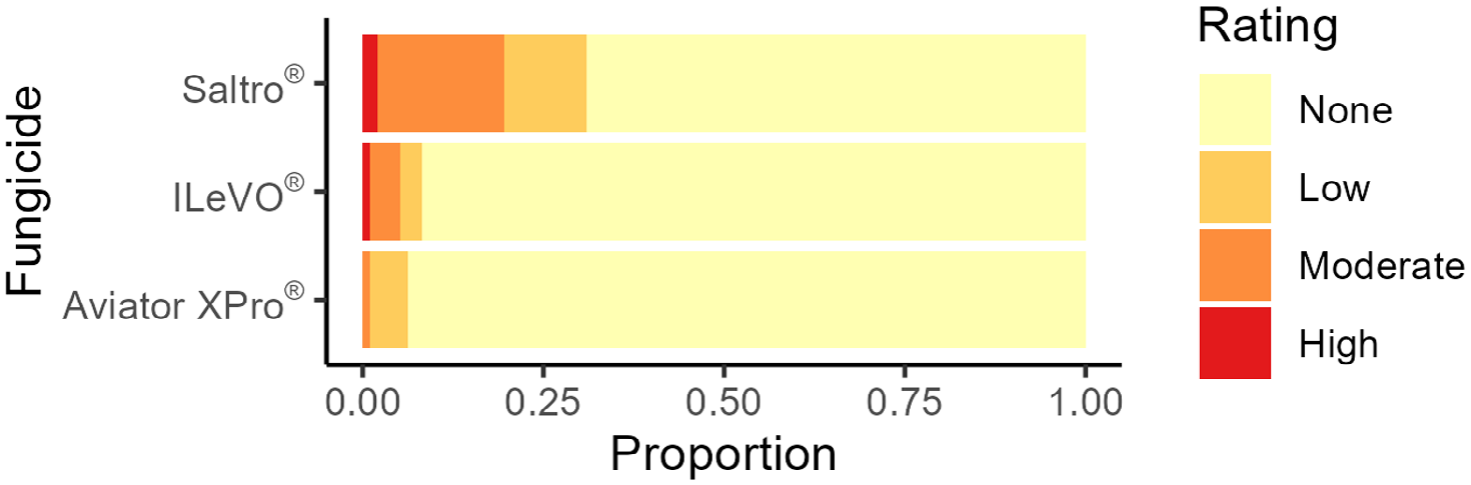
Fungicide disease rating for Saltro (pydiflumetofen), ILeVO (fluopyram) and Aviator XPro (bixafen + prothioconazole) in 2024 field sensitivity survey. Ratings are determined by the resistance score (RS). Each *Leptosphaeria maculans* population was categorised into high resistance (RS ≥ 0.5), moderate resistance (0.1 ≤ RS < 0.5), low resistance (0 < RS < 0.1) and no resistance (RS = 0).

### 2.2 Identification of SDHI Resistant Isolates

To determine whether isolates harbouring SDHI resistance were present in the populations identified as moderate or high disease ratings in the in planta assay, isolates were cultured from lesions. Following surface sterilisation, infected cotyledons were placed on media containing ampicillin and pydiflumetofen (100 ng mL^−1^) and single‐spore cultures were established from colonies that grew. Six isolates were cultured from five different populations (Table 2).

**TABLE 2:** Details of the six *Leptosphaeria maculans* isolates collected from the fungicide resistance survey and two susceptible isolates.

Isolate	Population	Chemistry cultured from	Location	Pydiflumetofen		Fluopyram		Bixafen		Fluxapyroxad		Mutations	
				EC_50_ ^a^	RF^b^	EC_50_	RF	EC_50_	RF	EC_50_	RF	SdhB	SdhD
D2^c^	N/A	N/A		3.1	0.6	70	1.3	1.3	0.4	31	1.2	WT	WT
D3^c^	N/A	N/A		2.3	0.5	35	0.6	0.9	0.3	17	0.6	WT	WT
24BL105	9099	Saltro	Cockaleechie, SA	45	9.6	53	1.0	36	13	> 1000	> 37	H277Y	WT
24BL106	9100	ILeVO	Wanilla, SA	264	54	> 1000	> 18	126	47	> 1000	> 37	H277L	WT
24BL107	9101	Saltro	Wanilla, SA	25	5.3	45	0.8	21	7.8	> 1000	> 37	H277Y	WT
24BL108	9103	Saltro	Edillilie, SA	502	103	> 1000	> 18	227	84	> 1000	> 37	H277L	WT
24BL109	9103	ILeVO	Edillilie, SA	> 1000	> 212	> 1000	> 18	130	48	> 1000	> 37	H277L	WT
24BL110	9104	Saltro	Wanilla, SA	945	193	> 1000	> 18	> 1000	> 370	> 1000	> 37	H277L	D144G

^a^ EC_50_ (ng mL^−1^) is the effective concentration for 50% inhibition of SDHI fungicides.

^b^ RF is resistance factor.

^c^ D2 (IBCN15) and D3 (IBCN16) were used as susceptible controls and were isolated in 1988 (Purwantara et al. 2000).

The in vitro sensitivity of six isolates was determined toward four SDHI actives (pydiflumetofen, fluopyram, bixafen, fluxapyroxad) using a mycelial growth method. Isolates were cultured onto V8 media amended with increasing concentrations of fungicides. Radial growth was measured after 5 days and an EC_50_ was calculated for each isolate and fungicide. Estimation of EC_50_ values for isolates that were not inhibited by > 50% at the maximum concentration (1000 ng mL^−1^) is unlikely to be accurate due to extrapolation of data and have been given a value of > 1000 ng mL^−1^. From Table 2, EC_50_ values for pydiflumetofen varied from 25 to > 1000 ng mL^−1^, fluopyram varied from 45 to > 1000 ng mL^−1^, bixafen varied from 21 to > 1000 ng mL^−1^ and fluxapyroxad all > 1000 ng mL^−1^. It was evident there were two distinct phenotype groups within the isolates. Isolates 24BL105 and 24BL107 were similar in that they both had high levels of resistance to fluxapyroxad, intermediate resistance to pydiflumetofen and bixafen but were susceptible to fluopyram, whereas isolates 24BL106, 24BL108, 24BL109 and 24BL110 were all highly resistant to all four SDHI chemistries. All isolates were also screened for resistance to the DMI fungicides, with no resistance detected (data not shown).

In planta sensitivities to SDHI fungicides were also assessed for the SDHI‐resistant isolates listed in Table 2. Results for two resistant isolates (24BL107, 24BL110) are presented, representing two resistance phenotypes, with SDHI‐sensitive isolate D2 included as a sensitive control. Canola seedlings were treated with eight fungicides (Table 1), either as a seed treatment (Saltro [pydiflumetofen, SDHI] and ILeVO [fluopyram, SDHI]) or foliar application (Miravis [pydiflumetofen, SDHI], Xzemplar [fluxapyroxad, SDHI], Aviator XPro [bixafen + prothioconazole, SDHI + DMI], Revystar [fluxapyroxad + mefentrifluconazole, SDHI + DMI], Proviso [prothioconazole, DMI] and Belanty [mefentrifluconazole]) applied 8 days after sowing. A formulated bixafen‐only product was not available for testing. The DMI fungicides Proviso (prothioconazole) and Belanty (mefentrifluconazole) were included as controls to confirm the sensitivity to the DMI chemistries.

Consistent with in vitro EC_50_ results, isolates 24BL107 and 24BL110 exhibited distinct sensitivity phenotypes in planta (Figure 2). Isolate 24BL107 caused high disease on the untreated plants and Xzemplar (fluxapyroxad) treatment, moderate disease on the Saltro and Miravis treatments (both pydiflumetofen) and no disease on the ILeVO (fluopyram) treatment. In contrast, isolate 24BL110 caused high levels of disease on the untreated plants and across all four fungicides, with no treatment causing any significant decrease in pathogenicity. The different resistance profiles observed across isolates in the in vitro and in planta screens indicated the high likelihood of at least two mutations being involved. All fungicide treatments that contained a DMI (Aviator XPro [bixafen + prothioconazole], Revystar [fluxapyroxad + mefentrifluconazole], Proviso [prothioconazole] and Belanty [mefentrifluconazole]), in either a mixture or solo product, controlled disease for all isolates, indicating these isolates do not have any resistance to the DMI chemistries. As expected, the sensitive isolate (D2) produced high levels of disease on the untreated plants and no disease on any of the fungicide treatments.

**FIGURE 2:**
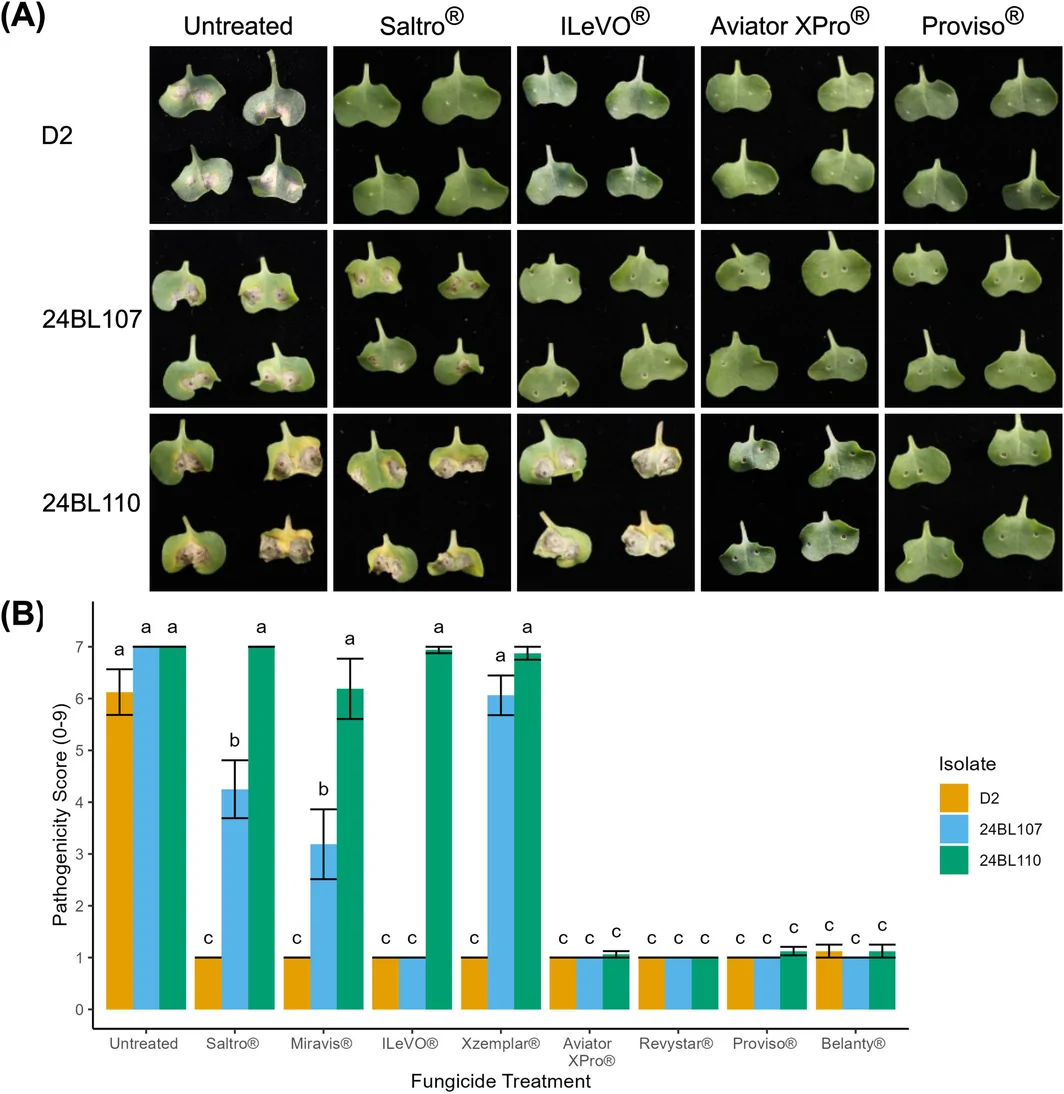
In planta fungicide sensitivity of D2, 24BL107 and 24BL110 toward the fungicides (Table 1). Fungicides used were Saltro (pydiflumetofen, seed dressing), Miravis (pydiflumetofen, foliar), ILeVO (fluopyram, seed dressing), Xzemplar (fluxapyroxad, foliar), Aviator XPro (prothioconazole and bixafen, foliar), Revystar (mefentrifluconazole and fluxapyroxad, foliar), Proviso (prothioconazole, foliar) and Belanty (mefentrifluconazole, foliar). (A) shows images of cotyledon lesions on selected fungicides and (B) the cotyledon pathogenicity scores for all fungicides. Different letters indicate results that are significantly different based on Tukey's Honestly Significant Difference test (*n* = 8, *α* = 0.05).

### 2.3 Crossing and Genetic Analysis Identified Mutations in the *SdhB* and *SdhD* Genes That Confer SDHI Resistance

Two crosses followed by bulked segregant analysis (BSA) on the progeny were used to map the underlying genetic factors conferring the different SDHI resistance phenotypes. The resistant isolates 24BL107 and 24BL110, which had differing SDHI sensitivity profiles, were crossed with sensitive isolates of the opposite mating type to create two different mapping populations. Progeny were phenotyped for their sensitivity to SDHI actives in vitro, then bulked into resistant and susceptible populations and sequenced. The BSA reads were analysed and quantitative trait loci (QTLs) identified.

For the D2 × 24BL107 cross, 128 progeny segregated into resistant (*n* = 65) and susceptible (*n* = 63) classes on pydiflumetofen, consistent with a single‐gene inheritance pattern (1:1 ratio, *p* = 0.86; Figure 3). BSA identified a significant QTL on supercontig SC00 associated with SDHI resistance (Figure 4, Table S1). For the D3 × 24BL110 cross, 122 progeny were screened for SDHI sensitivity. Initially, the progeny were screened against pydiflumetofen and segregated into three groups (highly resistant, intermediate resistant, susceptible) at a ratio of approximately 1:2:1, indicating the likely presence of two genes. It was hypothesised that the mutation in 24BL107 in combination with another mutation gave rise to resistance to fluopyram, and so fluopyram could be used to further segregate the progeny in the intermediate resistant group. All progenies were then rescreened for their responses against pydiflumetofen and fluopyram, and classified into four phenotypic groups: susceptible to both fungicides (SS, *n* = 37), resistant to pydiflumetofen only (IS, *n* = 25), intermediate resistant to both fungicides (II, *n* = 32) and resistant to both fungicides (RR, *n* = 28; Figure 5). Segregation was consistent with two unlinked genes (1:1:1:1 ratio, *p* = 0.45). BSA identified significant QTLs associated with SDHI resistance on SC00, SC03 and SC07 (Figure 4, Table S1).

**FIGURE 3:**
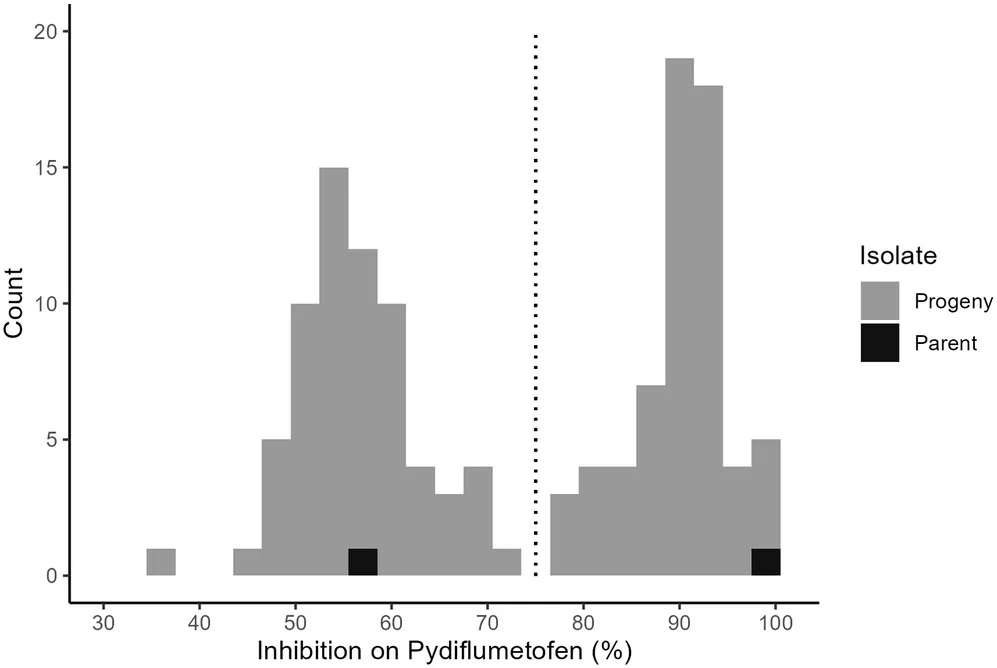
In vitro SDHI sensitivity assay categorises progenies from D2 × 24BL107 cross. The progenies are in light grey and parent isolates in dark grey. Isolates with > 75% inhibition (indicated with dotted vertical line) were categorised susceptible (*n* = 63) and < 75% inhibition were categorised as resistant (*n* = 65). All isolates were screened on untreated media and pydiflumetofen (100 ng mL^−1^).

**FIGURE 4:**
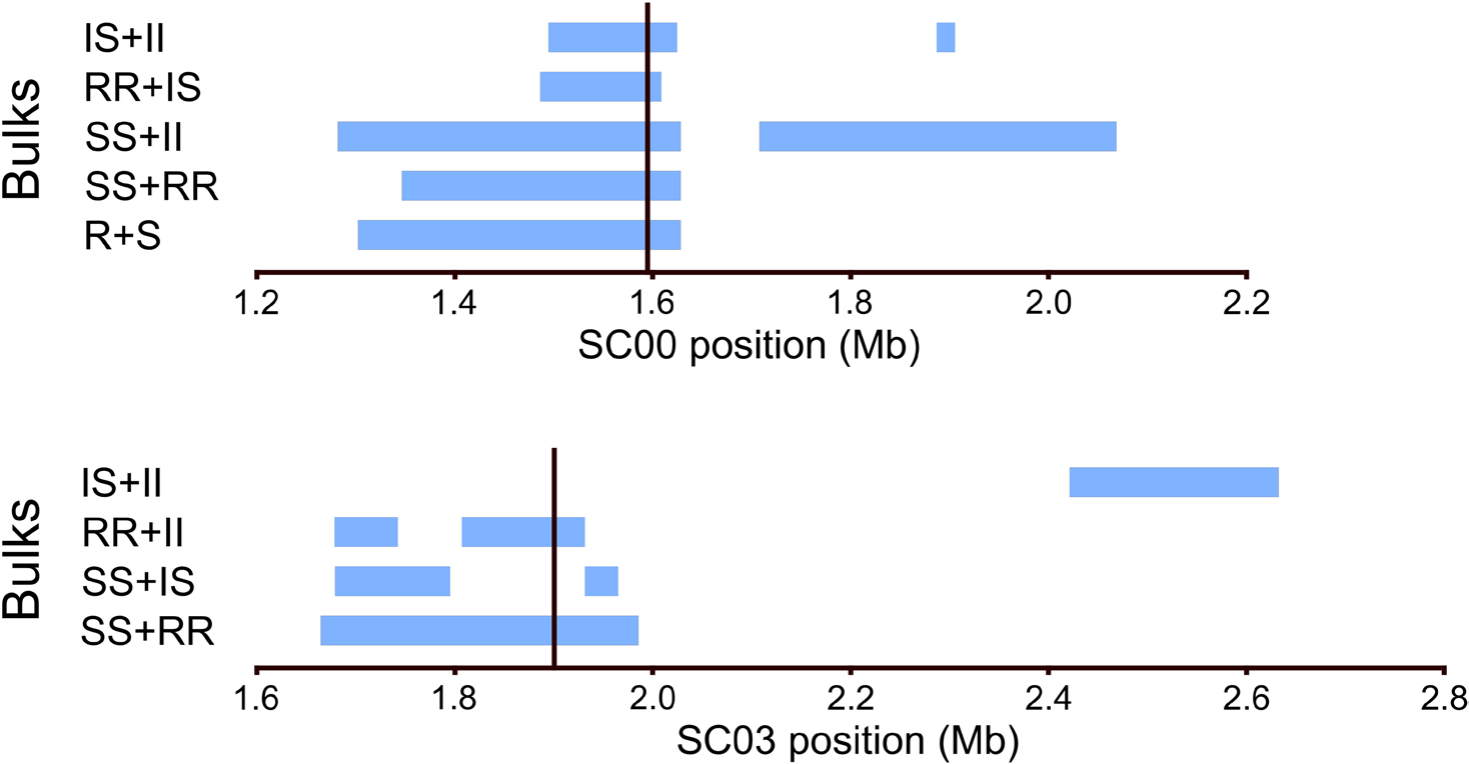
Locations of QTLs associated with SDHI resistance are on supercontigs SC00 (top) and SC03 (bottom). Each QTL is shown in the blue boxes and the vertical black lines indicate the positions of *SdhB* (top) and *SdhD* (bottom).

**FIGURE 5:**
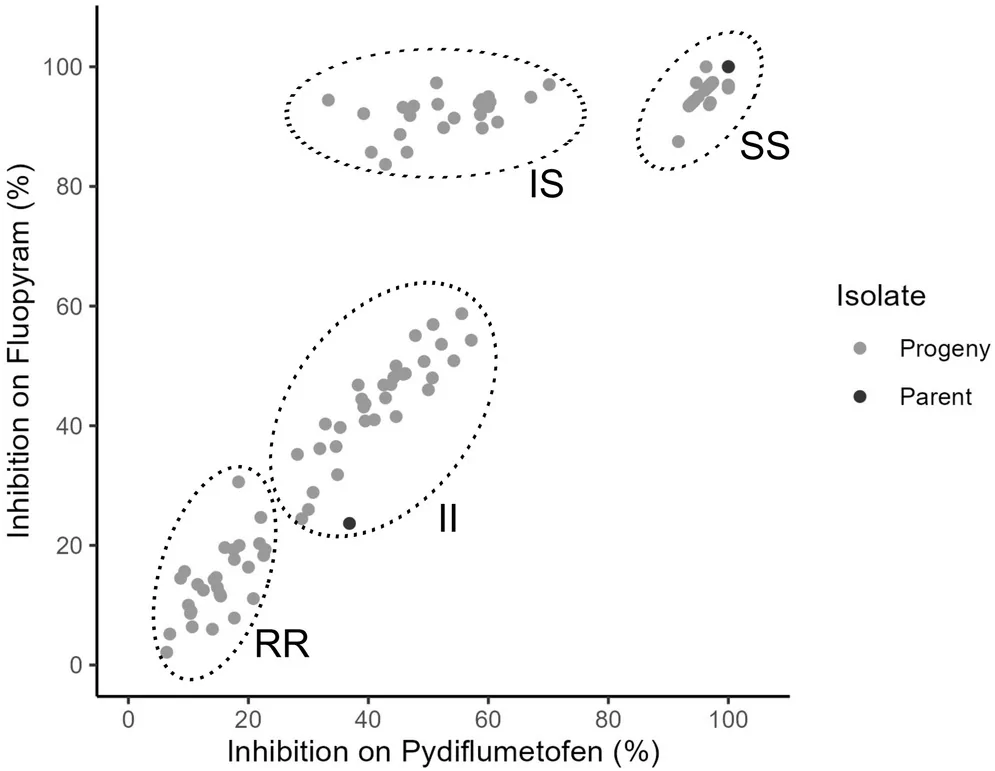
In vitro SDHI sensitivity assay categorises progenies from D3 × 24BL110 cross into four groups. Progenies are light grey points, and parental isolates are dark grey points. Progeny on top right were categorised into SS (susceptible to both pydiflumetofen and fluopyram; *n* = 37), top middle group was IS (intermediate resistant to pydiflumetofen and susceptible to fluopyram; *n* = 25), middle group was II (intermediate resistant to both pydiflumetofen and fluopyram; *n* = 32) and bottom left group was RR (resistant to both pydiflumetofen and fluopyram; *n* = 28). All progeny and parents were screened on pydiflumetofen (100 ng mL^−1^) and fluopyram (1000 ng mL^−1^).

The SC00 QTL identified from the D2 × 24BL107 cross overlapped with the QTLs identified in the D3 × 24BL110 cross. There were 24 SNPs that were significant across all five SC00 QTLs. The *SdhB* gene lies within this region, and across both crosses there were three significant SNPs in this gene. The *SdhB* gene was then sequenced using Sanger technology for the six resistant isolates and eight susceptible isolates (Table S2): four SNPs that differed from the JN3 strain reference sequence were found. The first SNP resulted in a T156A mutation that is not associated with resistance as it was also present in susceptible isolates; the second was a H277Y mutation present in 24BL105 and 24BL107; the third was a H277L mutation present in isolates 24BL106, 24BL108, 24BL109 and the fourth mutation in 24BL110 was synonymous and present in all isolates (Table 2). Within the four significant QTL regions located on SC03 there were no SNPs that were significant in every QTL, but there were four SNPs that were significant in three QTLs. The *SdhD* gene was identified in this region and is also the likely source of mutations causing SDHI resistance with one of these significant SNPs located in this gene. Sequencing of the *SdhD* gene region of the same eight isolates as above revealed a SNP in S*dhD* resulting in a D144G substitution for 24BL110, which also has the *SdhB*‐H277L mutation, as the only mutation in any of the isolates sequenced. Only a single synonymous mutation was detected in the sequencing of the *SdhC* gene region. Two QTLs were also identified on SC07, and further work would be required to identify a candidate gene.

Kompetitive allele‐specific PCR (KASP) markers were developed for all three SNPs associated with SDHI resistance to aid in the analysis of fungal populations. The markers were used on progenies to match the genotype with resistance phenotype for *SdhB*‐H277Y, *SdhB*‐H277L and *SdhD*‐D144G (Figure S1). For the D2 × 24BL107 cross, the KASP generated genotype (*SdhB*‐H277Y) aligned with the pydiflumetofen‐resistant phenotype for 96.7% of the progeny. For the D3 × 24BL110 cross, the KASP results were accurate to the phenotype groups for 88.6% of progeny with four of the mismatches between the II and RR groups. Phenotypic classification of the II and RR classes is not clear cut with some phenotypic overlap (Figure 5), therefore, the classification of some progeny may be incorrect.

### 2.4 SDHI Resistance by *Sdh* Mutations Was Validated by Gene Complementation Experiments

To validate whether the *Sdh* gene mutations, that is, *SdhB*‐H277Y, *SdhB‐*H277L and *SdhD*‐D144G, confer SDHI resistance, two‐way genetic complementation assays were conducted (Figure 6). These experiments were conducted within the context that the *Sdh* genes are essential for viability, so they cannot be deleted and then replaced with different alleles. Five complementation constructs were assembled: two contained the susceptible alleles (pLmSdhB‐WT and pLmSdhD‐WT) and three contained the resistant alleles (pLmSdhB‐H277Y, pLmSdhB‐H277L and pLmSdhD‐D144G). In two‐way complementation assays, susceptible‐allele constructs were transformed into resistant isolates (pLmSdhB‐WT for 24BL105 with *SdhB*‐H277Y, pLmSdhB‐WT for 24BL106 with *SdhB*‐H277L and pLmSdhD‐WT for 79R103, which is a progeny from crossing with *SdhD*‐D144G) and resistant‐allele constructs were transformed into susceptible isolates (pLmSdhB‐H277Y/pLmSdhB‐H277L/pLmSdhD‐D144G for D3 with wild‐type alleles). These transformants have ectopic integration of the T‐DNAs and thus contain both the endogenous copy of the *Sdh* gene and the transformed *SdhB/D* allele, allowing for the expression and interaction of both alleles to be observed. Quantitative PCR (qPCR) and KASP analyses confirmed expression of both the endogenous and introduced *Sdh* alleles in all transformants (Figures S2 and S3; Tables [Link], [Link], [Link]).

**FIGURE 6:**
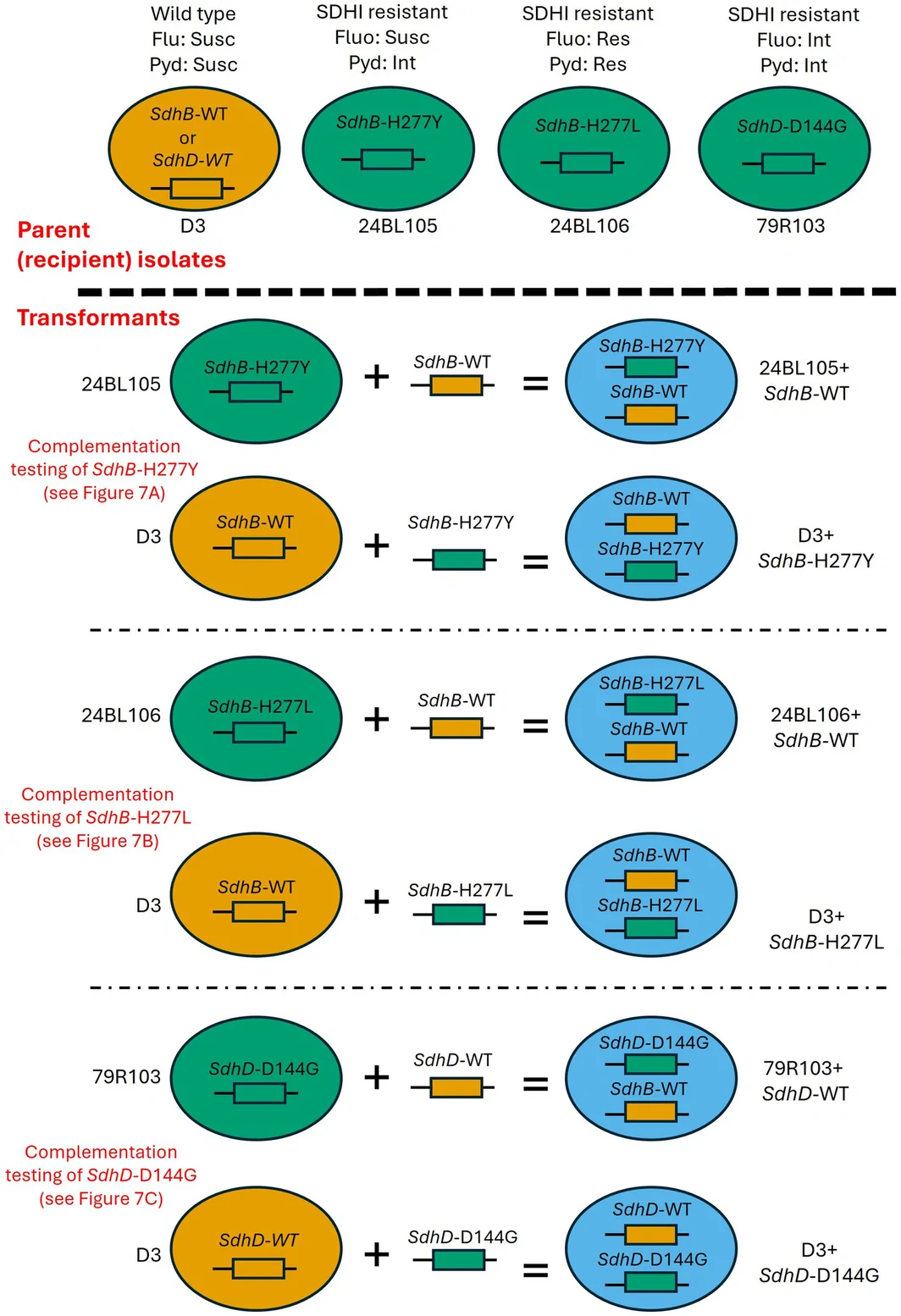
Graphical representation of the genetic complementation strategy to define the action of mutations in *Sdh* genes in conferring sensitivity or resistance against SDHI fungicides. The strategy was used to test the role of the *SdhB*‐H277Y, *SdhB*‐H277L and *SdhD*‐D144G mutations in resistance to the fluopyram (Flu) and pydiflumetofen (Pyd) chemistries. Above the dashed line, parent isolates and their resistance types toward fluopyram and pydiflumetofen gained from Section 2.2 Table 2 are shown. Int, intermediate; Res, resistant; Susc, susceptible. Transformants' in vitro sensitivity is presented in Figure 7.

The sensitivity to pydiflumetofen and fluopyram was determined in vitro for 18 transformants and the parent isolates. Isolates were cultured on V8 medium with increasing concentrations of fungicides. Radial growth was measured after 7 days, and EC_50_ values were estimated. Each complementation type had three biological replicates: D3 + *SdhB*‐H277Y, D3 + *SdhB*‐H277L, D3 + *SdhD*‐D144G, 24BL105 + *SdhB*‐WT, 24BL106 + *SdhB*‐WT and 79R103 + *SdhD*‐WT (Figure 7). Introduction of the susceptible allele (*SdhB*‐WT) significantly reduced pydiflumetofen resistance in 24BL105 (24BL105 + *SdhB*‐WT) by 3.6‐fold and reduced both pydiflumetofen and fluopyram resistance in 24BL106 (24BL106 + *SdhB*‐WT) by 5.9‐ and 5.1‐fold, respectively. Conversely, introduction of *SdhB*‐H277L allele into the susceptible background (D3 + *SdhB*‐H277L) significantly increased resistance to pydiflumetofen and fluopyram by 9.0‐ and 9.5‐fold, respectively, whereas *SdhB*‐H277Y (D3 + *SdhB*‐H277Y) had no significant effect on pydiflumetofen resistance and was associated with reduced fluopyram resistance. Introduction of susceptible allele (*SdhD*‐WT) into 79R103, (79R103 + *SdhD*‐WT) significantly reduced pydiflumetofen and fluopyram resistance by 3.2‐ and 2.2‐fold, respectively, while introduction of *SdhD*‐D144G into D3 had no statistically significant effect on resistance. As the ectopic transformants retained their endogenous *Sdh* allele together with the introduced allele, transformants derived from susceptible and resistant parental backgrounds displayed different in vitro sensitivity profiles. Together these complementation assays validated *SdhB*‐H277Y, *SdhB*‐H277L and *SdhD*‐D144G as determinants of SDHI resistance in *L. maculans*.

**FIGURE 7:**
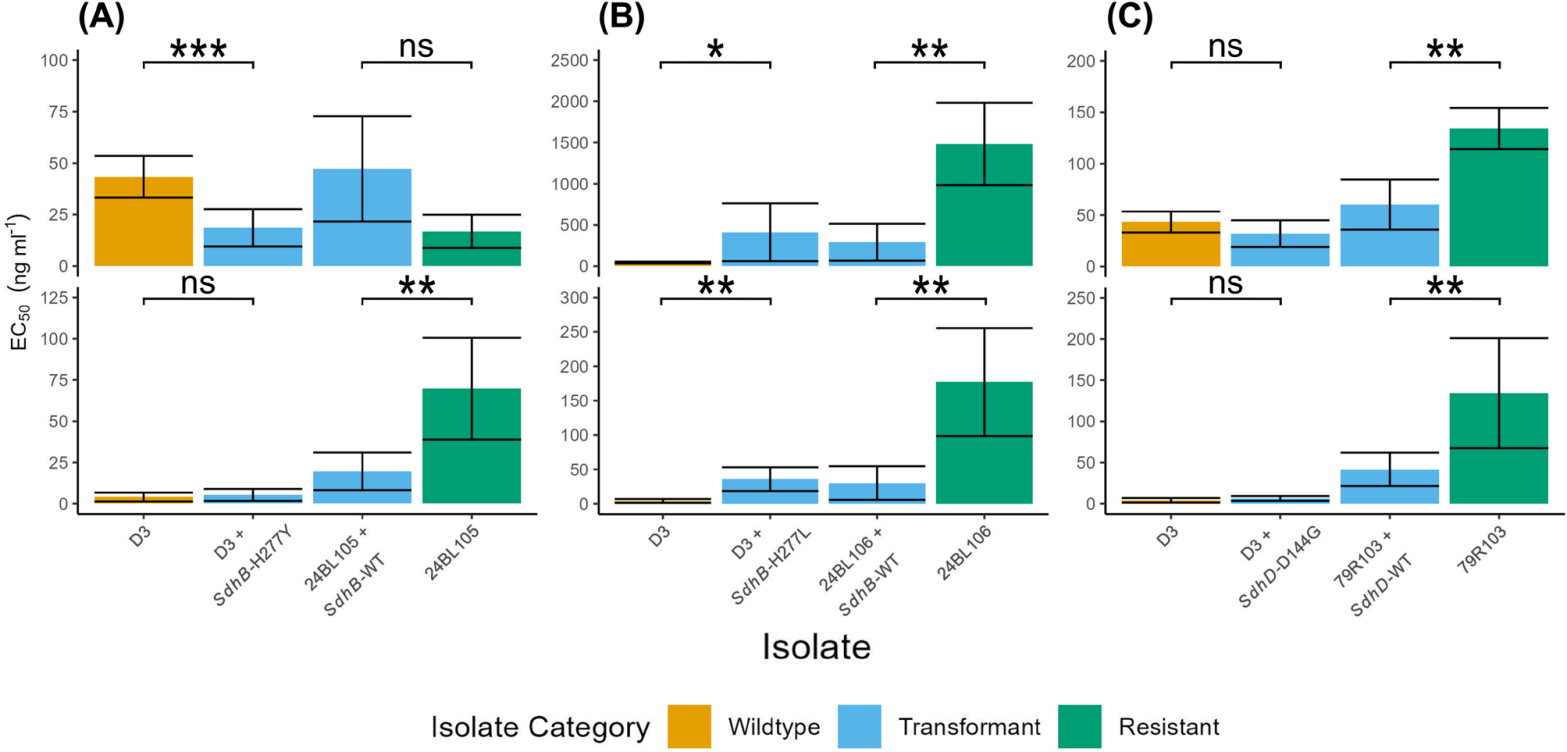
In vitro sensitivity of wild‐type (D3), resistant (24BL105, 24BL106 and 79R103) and transformant isolates toward fluopyram (top row) and pydiflumetofen (bottom row). Sensitivity given as EC_50_ and is the concentration where mycelial growth is reduced by 50%. Significant differences based on Student's *t*‐test (*n* = 3, except for 79R103 treated with pydiflumetofen and all parent isolates treated with fluopyram *n* = 2). Significance: ns, not significant; *p ≤ 0.05, **p ≤ 0.01, ***p ≤ 0.001. Complementations involving *SdhB*‐H277Y (A), *SdhB*‐H277L (B) and *SdhD*‐D144G (C). Representative visual examples of plate image of in vitro sensitivity assay are shown in Figure S4.

### 2.5 Identification of the Frequency of *Sdh* Gene Mutations in Field Populations by Deep Amplicon Sequencing Assay

A calibrated deep amplicon sequencing (DAS) assay was conducted to identify the frequency of *Sdh* mutations in populations (Figure S5). Ascospore populations were liberated from 110 stubble samples from a 2024 fungicide resistance field survey, of which nine populations failed to release sufficient spores and were not analysed. The *SdhB* and *SdhD* genes were amplified from each ascospore population and the resulting amplicons sequenced. Analysis of the sequencing data revealed four SNPs in each gene region and estimated the frequency of each mutation (Table 3). Resistance‐associated mutations included *SdhB*‐H277Y, *SdhB*‐H277L and *SdhD*‐D144G, while the remaining variants were synonymous, non‐coding or not associated with resistance. The frequencies of these three mutations were combined to estimate overall SDHI resistance frequency within each population. As DAS cannot determine whether multiple mutations occur within the same individual isolate, co‐occurrence of *SdhB* and *SdhD* mutations could not be assessed (i.e., 24BL110). Fifty‐nine of the populations had resistance frequencies below 0.1%, 37 between 0.1% and 1% and only five exceeded 1%. The results from the DAS assay were compared to the fungicide resistance survey results to determine if there is a correlation between the frequency of SDHI resistant alleles in the population and the resistance scores (RS) previously determined. There was a significant, positive correlation between the frequency of the *SdhB*‐H277L and the RS of Saltro (pydiflumetofen) (*p* = 0.0026, *ρ* = 0.308, Figure 8) and RS of ILeVO (fluopyram) (*p* = 0.029, *ρ* = 0.228, Figure 8). There were no significant correlations between the frequencies of *SdhB*‐H277Y or *SdhD*‐D144G and the RS of either Saltro (pydiflumetofen) or ILeVO (fluopyram).

**TABLE 3:** Details and the minimum, maximum and mean frequency of each mutation in *SdhB* and *SdhD*.

Gene	Position from start codon	Nucleotide change	Mutation	Minimum	Maximum	Mean
*SdhB*	—	—	Ref	0	17.4	0.7
	584	A→G	T156A	37.3	99.5	82.8
	1084	T→C	S1	70.7	100	98.1
	998	C→T	H277Y	0	20.4	0.6
	999	A→T	H277L	0	4.1	0.1
*SdhD*	—	—	Ref	30.1	99.9	85.9
	721	C→A	S1	0	0.9	0
	603	A→G	D144G	0	9.6	0.3
	1068	A→G	NC1	0	0.5	0.2
	1024	C→G	NC2	0	69.8	13.8

Abbreviations: NC, noncoding; Ref, reference; S, synonymous.

**FIGURE 8:**
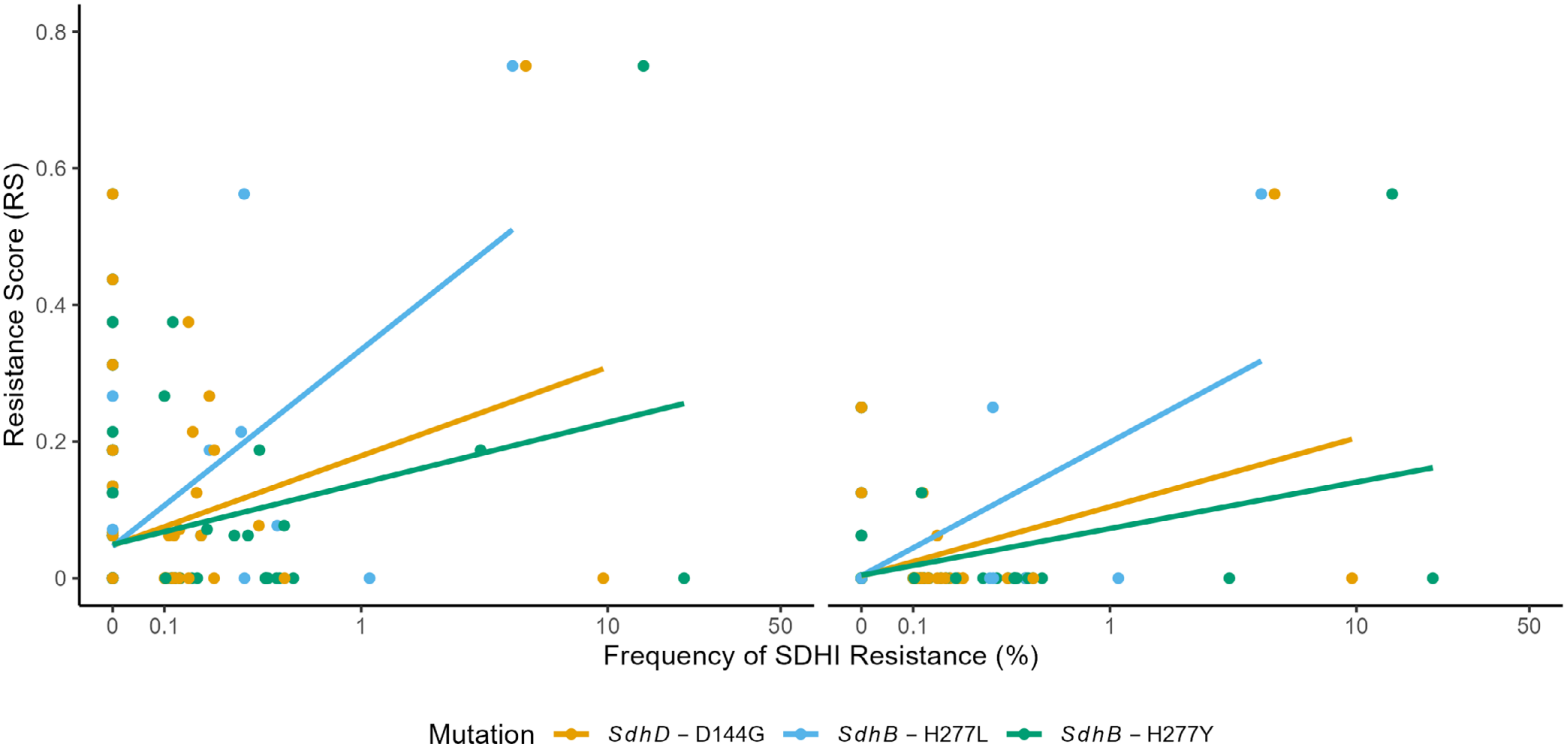
Relationships between frequency of mutations *SdhD*‐D144G (orange), *SdhB*‐H277L (blue) and *SdhB*‐H277Y (green) and resistance score (RS) for Saltro (pydiflumetofen) (left) and ILeVO (fluopyram) (right). The correlation method is described in Section 4.11.

## 3 Discussion

Fungicide applications are an integral component of blackleg management and the reliance on fungicides within canola industries has increased substantially worldwide. SDHI fungicides have been registered for blackleg control since 2016, and between 2018 and 2023 no Australian populations showed high levels of resistance to SDHI fungicides (McCallum, Idnurm, and Van de Wouw 2025). SDHI‐resistant isolates were detected for the first time in a 2024 Australian fungicide resistance survey, with 2.1% and 1.0% of populations being highly resistant to Saltro (pydiflumetofen) and ILeVO (fluopyram), respectively (Figure 1). Fungicide‐resistant isolates were collected, and the resistance‐associated mutations were identified through crosses and BSA genetic mapping and their role in conferring SDHI resistance was validated by transformation experiments. With these findings, molecular markers were developed and the frequencies of *Sdh* gene mutations in field populations were estimated.

Among six *L. maculans* isolates, two distinct SDHI‐resistant phenotypes were uncovered: one that was highly resistant to all four SDHI chemistries and the other that was highly resistant to fluxapyroxad, intermediately resistant to pydiflumetofen and bixafen and susceptible to fluopyram (Table 2, Figure 2). The BSA mapping revealed that SDHI resistance in *L. maculans* is primarily associated with target‐site mutations in the genes encoding SDHB and SDHD. Sanger sequencing of *SdhB* and *SdhD* from resistant isolates detected three SNPs: *SdhB*‐H277Y, *SdhB*‐H277L and *SdhD*‐D144G. These target site mutations have been previously observed in other plant pathogens. For example, *SdhB*‐H277Y/L mutations have been found in *Pyrenophora teres* (Rehfus et al. 2016; Mair et al. 2023), *Corynespora cassiicola* (Sun et al. 2022), *A. alternata* (Avenot et al. 2014; Yang et al. 2015; Förster et al. 2022) and *B. cinerea* (Hu et al. 2016; Lalève et al. 2014), while *SdhD*‐D144G has been detected in *P. teres* (Rehfus et al. 2016; Mair et al. 2023) and *Zymoseptoria tritici* (Fraaije et al. 2012). Similar phenotypes to this study have been observed in other pathogens. For example, the *SdhB*‐H272Y in *B. cinerea* and the *SdhB*‐H248Y in *Fusarium asiaticum* retained susceptibility to fluopyram despite being completely resistant to other SDHI fungicides (Avenot et al. 2014; Lalève et al. 2014; Song et al. 2026). Similarly, the *SdhB*‐H277L mutation results in complete resistance across all tested SDHI fungicides in *A. alternata* (*SdhB*‐H277L), *B. cinerea* (*SdhB*‐H272L), *Fusarium graminearum* (*SdhB*‐H248L) and *Ustilaginoidea virens* (*SdhB*‐H239L) (Lalève et al. 2014; Förster et al. 2022; Yin et al. 2024; Jie et al. 2025).

Two‐way complementation assays validated that *SdhB*‐H277Y, *SdhB*‐H277L and *SdhD*‐D144G confer SDHI resistance in *L. maculans*. Overall, the introduction of wild‐type alleles into resistant isolates reduced SDHI resistance, whereas introduction of mutant alleles into susceptible backgrounds increased resistance, supporting a direct role of these mutations in altering SDHI sensitivity. Among the mutations tested, *SdhB*‐H277L conferred the strongest resistance phenotypes. In contrast, introduction of *SdhB*‐H277Y into the susceptible isolate did not significantly increase pydiflumetofen resistance and resulted in hypersensitivity to fluopyram (Figure 7). This is consistent with previous reports in *Mycosphaerella graminicola* (*Zymoseptoria tritici*) (*SdhB*‐H267Y), *B. cinerea* (*SdhB*‐H272Y), *A. alternata* (*SdhB*‐H277Y) and *F. asiaticum* (*SdhB*‐H248Y), which have been associated with fluopyram hypersensitivity, possibly due to increased fluopyram binding efficiency (Scalliet et al. 2012; Leroux et al. 2010; Labourdette et al. 2011; Song et al. 2026). Fungicide‐specific resistance profiles have also been reported for other SDH protein modifications; for example, SdhC2‐A83V in *F. asiaticum* confers pydiflumetofen and fluopyram resistance but not resistance to benzovindiflupyr (Li et al. 2025). Together, these findings highlight that mutations within the same target gene can confer distinct resistance profiles across SDHI actives, emphasising the need to investigate cross‐resistance between individual active chemicals.

The role of *SdhD*‐D144G in SDHI resistance was supported by reduced resistance following introduction of the wild‐type allele, although introduction of *SdhD*‐D144G into a susceptible background did not significantly alter sensitivity to either fungicide (Figure 7). Together with the absence of increased pydiflumetofen resistance in the D3 (susceptible isolate) + *SdhB*‐H277Y transformants, these contrasting outcomes suggest different dominance relationships between alleles (Figure 7). *SdhB*‐WT appeared to partially suppress the effect of *SdhB*‐H277L and to be dominant over that of *SdhB*‐H277Y. Similarly, *SdhD*‐WT appeared to dominate the effect of *SdhD*‐D144G. This interpretation is consistent with previous work in which heterozygous *M. graminicola* transformants showed dominance relationships between wild‐type and mutated alleles (Scalliet et al. 2012). In this study, transformants were generated via ectopic integration of an additional gene copy rather than targeted gene replacement, because homologous recombination is highly inefficient in *L. maculans* and the succinate dehydrogenase complex is essential for viability. Consequently, the observed phenotypes should be interpreted as evidence supporting the role of these alleles in determining SDHI sensitivity, rather than as direct measures of allele function in a genetic background containing only the mutant/wild‐type allele.

The DAS assay provided a sensitive and effective approach for detecting and quantifying *Sdh* mutations in *L. maculans* field populations. The calibration standards for DAS demonstrated the reliable quantification between 0.1% and 100% of mutant allele frequency, although estimates below 0.1% should be interpreted cautiously due to signal plateauing (Figure S5). Despite this limitation, the application of the DAS assay to the 2024 survey revealed that SDHI resistance remained at low frequencies in most Australian populations, with only five populations exceeding 1% resistance. This represents the first detection of SDHI resistance in *L. maculans* field populations, with no resistance reported in 2018–2023 surveys (McCallum, Idnurm, and Van de Wouw 2025), suggesting that resistance is still emerging rather than widespread. However, the detection of SDHI resistance alleles across multiple populations, together with a significant positive correlation between resistance frequency and reduced SDHI sensitivity (Figure 8), indicates that *Sdh* mutations are associated with shifts toward SDHI resistance in field populations. Among the mutations identified, *SdhB*‐H277L showed the strongest association with reduced sensitivity and is therefore likely to have the greatest impact on field resistance. Notably, all populations with elevated SDHI resistance frequencies from the 2024 survey were located on the Eyre Peninsula of South Australia. This region was also the site of rapid breakdown of major resistance genes in 2004 (Sprague et al. 2006) and 2011 (Van de Wouw et al. 2014), highlighting the adaptive capacity of *L. maculans* and the importance of monitoring and integrated disease management.

Overall, this study provides the first evidence that SDHI resistance has emerged in *L. maculans* populations and identifies *SdhB* and *SdhD* mutations as causal factors. By combining genetic mapping, allele complementation and DAS assays, this work links *Sdh* mutations to altered SDHI sensitivity in both experimentally isolated strains and in field populations. Although SDHI fungicides were first registered for canola in Australia in 2016, widespread use began only in 2020–2021 with the introduction of fluopyram and pydiflumetofen seed treatments (McCallum, Idnurm, and Van de Wouw 2025; Van de Wouw, Scanlan, et al. 2021). The detection of resistance within 3–4 years of widespread adoption mirrors the rapid breakdown of genetic resistance bred into canola cultivars and is consistent with the high evolutionary potential of *L. maculans* to overcome mechanisms of control (Sprague et al. 2006; Van de Wouw et al. 2014). The occurrence of isolates carrying mutations in both the *SdhB* and *SdhD* genes further raises concerns about the long‐term durability of SDHI fungicides. Together, these findings highlight the importance of ongoing surveillance and proactive resistance management to preserve SDHI efficacy for blackleg control.

Importantly, SDHI resistance frequencies were low in most field populations, creating a critical opportunity to investigate the development of fungicide resistance and to take preventive actions. The molecular markers and surveillance framework established here can support early detection, annual monitoring and more informed fungicide stewardship before resistance becomes widespread. Future research should focus on refining functional validation of mutant alleles through gene expression analysis or enzyme activity assays, resolving additional loci that may contribute to SDHI resistance and tracking shifts in SDHI resistance frequency across growing seasons. Together, this work provides both a foundation for future research and a practical basis for proactive fungicide resistance management.

## 4 Experimental Procedures

### 4.1 Fungicide Resistance Survey

In 2024, 110 populations were screened for resistance to three commercially registered fungicides (Table 1), Aviator XPro (bixafen, prothioconazole), Saltro (pydiflumetofen) and ILeVO (fluopyram), using the previously developed in planta assay (Van de Wouw, Scanlan, et al. 2021). Saltro (pydiflumetofen) and ILeVO (fluopyram) were applied to canola seeds as per manufacturer instructions, and Aviator XPro (bixafen, prothioconazole) was applied as a foliar spray to 8‐day‐old cotyledons at label rate. The proportion of infected cotyledons was determined in each punnet and each treatment. Resistance score (RS) was calculated as the proportion of infected cotyledons in fungicide‐treated seedlings divided by the proportion of infected cotyledons in untreated seedlings for each fungicide treatment and population (Van de Wouw, Scanlan, et al. 2021). ‘High’ resistance was categorised as RS > 0.5, ‘moderate’ resistance was an RS between 0.1 and 0.5, ‘low’ resistance was between 0 and 0.1 and an RS of 0 is ‘none’ resistance.

### 4.2 Isolate Collection

During the fungicide resistance survey, isolates were collected from lesions that formed on fungicide‐treated plants. Lesions were excised from cotyledons and surface sterilised with a 10% bleach solution before being rinsed twice in sterile water. The infected plant materials were then placed onto V8 medium (10% Campbell's V8 juice, 2% agar, 0.375% calcium carbonate) amended with ampicillin and pydiflumetofen (100 ng mL^−1^). Individual pycnidia were removed from these cultures and placed onto fresh V8 medium amended with pydiflumetofen. Cultures that grew at this stage were deemed likely to be resistant and retained for further analysis. All cultures were maintained at 22°C under a 12‐h light/12‐h dark photoperiod.

### 4.3 In Vitro Sensitivity Assay

Sensitivity of six isolates collected from the fungicide resistance survey and two susceptible isolates (Table 2) was assessed against four SDHI chemistries (pydiflumetofen, bixafen, fluopyram and fluxapyroxad). The fungicide actives were stored in 10 mg mL^−1^ aliquots dissolved in 90% dimethyl sulphoxide (DMSO)/10% ethanol at 4°C until use. Fungicide‐amended V8 agar was prepared using serial dilutions (0.1–1000 ng mL^−1^, 10‐fold dilution), and isolates were cultured on amended media using plugs (5 mm diameter) taken from the actively growing hyphal margin of 7‐day‐old cultures. After 5 days of incubation, radial growth was measured. The growth of each culture was determined by subtracting the plug diameter from the average culture diameter. EC_50_ values were estimated by fitting dose–response curves using the *drc* v. 3.0 package in R v. 4.4.2 (R Core Team 2024; Ritz et al. 2015). This assay for six isolates had two biological replicates and two technical replicates.

The sensitivity of complemented transformants to pydiflumetofen and fluopyram was determined using the same approach. Nine concentrations of pydiflumetofen (0, 5, 10, 20, 40, 80, 150, 300, 600 ng mL^−1^) and fluopyram (0, 10, 20, 40, 80, 160, 320, 640, 1280 ng mL^−1^) were made for 18 complemented transformants: D3 + *SdhB*‐H277Y, D3 + *SdhB*‐H277L, D3 + *SdhD*‐D144G, 24BL105 + *SdhB*‐WT, 24BL106 + *SdhB*‐WT and 79R103 + *SdhD*‐WT (three biological replicates for each complementation) and the parent isolates (D3, 24BL105, 24BL106 and 79R103 [which is a progeny of the D3 × 24BL110 cross with the mutation *SdhD*‐D144G], three biological replicates) with two technical replicates for each isolate (79R103 for pydiflumetofen and all parental isolates for fluopyram have two biological replicates due to the shortage of chemicals). Cultures were grown on fungicide‐amended V8 agar for 7 days, after which radial growth was measured (representative visual examples are provided in Figure S4). EC_50_ values were estimated using the AAT Bioquest EC_50_ calculator (AAT Bioquest 2026). R Statistical Software v. 4.4.2 (R Core Team 2024) was used to conduct pairwise *t‐*tests separately for each fungicide. Each comparison was conducted within a matching group, including the parental isolate versus its transformant (e.g., D3 vs. D3 + *SdhB*‐H277Y and 24BL105 vs. 24BL105 + *SdhB*‐WT).

### 4.4 In Planta Sensitivity Assay

Two representative SDHI‐resistant isolates collected from the fungicide resistance survey and the susceptible control isolate, D2, (Table 2) were screened in planta to confirm resistance. Pycnidiospores were collected from each isolate for inoculation. Briefly, plugs were taken from 10‐day‐old cultures, spread onto fresh V8 medium and left to grow for 12 days. Plates were then flooded with sterile water and scraped with a scalpel to free the spores. The spore solution was filtered using Miracloth, diluted to a concentration of 1 × 10^7^ spores mL^−1^ and then stored in 5 mL aliquots at −20°C until use.

Canola seed (cv. ATR‐Stingray) was sown either fungicide treated or untreated, and grown in a glasshouse at 22°C. This cultivar was used as it has no effective genetic resistance and therefore all isolates can cause disease in the absence of fungicide. Eight‐day‐old seedlings were treated with foliar fungicides at manufacturer's label rate. Details of the eight fungicides used are shown in Table 1. Inoculations occurred at least 24 h after foliar fungicide treatments. Each lobe of every cotyledon was wounded using tweezers then sprayed with suspensions of 1 × 10^6^ spores mL^−1^ using a Preval Sprayer (https://preval.com/diy‐pro‐products/preval‐sprayer/) until run‐off. Disease severity was measured after 12–14 days on each cotyledon lobe as a 0–9 pathogenicity score as previously described (Koch et al. 1991; Marcroft et al. 2012).

Statistical analysis and graphics were completed using the *tidyverse* package in R v. 4.4.2 (R Core Team 2024; Wickham et al. 2019). Disease severity responses to fungicides were analysed by ANOVA, followed by Tukey's honestly significant difference test (*α* = 0.05) for multiple comparisons. Each fungicide × isolate combination included eight replicate plants (R Core Team 2024; de Mendiburu and Yaseen 2020).

### 4.5 Bulked Segregant Analysis

Two segregating populations were generated through in vitro crosses between D2 × 24BL107 and D3 × 24BL110 following (Balesdent et al. 2001). Parent mating types were first confirmed by PCR using primers in Table 4 (Cozijnsen and Howlett 2003). A total of 128 progeny was obtained from D2 × 24BL107 and 122 progeny from D3 × 24BL110. All progeny and parents were phenotyped on a discriminative dose of pydiflumetofen (100 ng mL^−1^) and cross D3 × 24BL110 was further screened on fluopyram (1000 ng mL^−1^). Agar plugs were taken from 10‐day‐old colonies and transferred onto V8 media amended with a fungicide or untreated. Cultures were left to grow for 5 days before the diameter of the culture was measured in two perpendicular directions. To determine culture growth, the plug diameter (5 mm) was subtracted from the average culture diameter, and the formula was used to calculate an inhibition rate:

$$\text{Inhibition}\;\left(\%\right)=\frac{\text{Growt}h^{\text{Untreated}}-\text{Growt}h^{\text{Treated}}}{\text{Growt}h^{\text{Untreated}}}\times 100$$

The inhibition rate was used to categorise the progeny into bulks with similar phenotypes. Cross D2 × 24BL107 was split into a susceptible (S, inhibition > 75%) and resistant (R, inhibition < 75%) bulk. Cross D3 × 24BL110 was split into four bulks: susceptible to both (SS, pydiflumetofen and fluopyram inhibition > 75%), intermediate resistant to pydiflumetofen and susceptible to fluopyram (IS, pydiflumetofen inhibition < 75% and fluopyram inhibition > 75%), intermediate resistance to both (II, pydiflumetofen inhibition 25%–75% and fluopyram inhibition 35%–75%) and full resistance to both (RR, pydiflumetofen inhibition < 25% and fluopyram inhibition < 35%). A chi‐square test was used to determine if the crosses were segregating as expected for a single gene or two unlinked genes using the *chisq.test()* function in R v. 4.4.2 (R Core Team 2024).

**TABLE 4:** Details of primers used.

Primer	Sequence	Tag	Description	References
MAT 1‐1	CTCGATGCAATGTACTTGGAGC	N/A	Detection of mating type MAT1‐1	Cozijnsen and Howlett (2003)
MAT 1‐2	CGGAGGTGAAGTTGAAGCCG	N/A	Detection of mating type MAT1‐2	Cozijnsen and Howlett (2003)
MAT idiom	TGGCGAATTAAGGGATTGCTG	N/A	Common primer for detection of mating type	Cozijnsen and Howlett (2003)
KASP_SDHB_277L_Res	CGAGCAGTTCAAGATGGTGA	FAM	KASP	This study
KASP_SDHB_277L_Sus	CGAGCAGTTCAAGATGGTGT	HEX	KASP	This study
KASP_SDHB_277L_Com	GGATGCCCTCAACAACAGC	N/A	KASP	This study
KASP_SDHB_277Y_Res	CAACAGCATGAGCTTGTACCGCTGCT	FAM	KASP	This study
KASP_SDHB_277Y_Sus	CAACAGCATGAGCTTGTACCGCTGCC	HEX	KASP	This study
KASP_SDHB_277Y_Com	GGGCAAGTCCTCGAGCAGTT	N/A	KASP	This study
KASP_SDHD_Res	CAGGGCGTGTATCATCGG	FAM	KASP	This study
KASP_SDHD_Sus	CAGGGCGTGTATCATCGA	HEX	KASP	This study
KASP_SDHD_Com	CAGCAGCGCGAATCTTGG	N/A	KASP	This study
MAI0247	CCGTGCAGTCTTTCCTTC	N/A	qPCR for housekeeping gene (*Actin*)	Van de Wouw et al. (2017)
MAI0248	TTTTCCATGTCATCCCAG	N/A	qPCR for housekeeping gene (*Actin*)	Van de Wouw et al. (2017)
qSdhB_F1	CCTGACATGACGCTCTTCTAC	N/A	qPCR for *SdhB*	This study
qSdhB_R1	TCTTCCTTGGTTTGACGGTATT	N/A	qPCR for *SdhB*	This study
qSdhD_F1	CTGTACCCAACCCAGATTACAC	N/A	qPCR for *SdhD*	This study
qSdhD_R1	CGATGGTCAAGGGAATCAGAC	N/A	qPCR for *SdhD*	This study
LmsdhB‐1F	CGGTCCAACTCGATGATTGC	N/A	Sanger sequencing and deep amplicon sequencing (DAS) for *SdhB*	McCallum, Idnurm, and Van de Wouw (2025)
LmsdhB‐1R	TGATGAAAAAGAATTTGTGACTCGC	N/A	Sanger sequencing and DAS for *SdhB*	McCallum, Idnurm, and Van de Wouw (2025)
LmSdhC‐1R	CGGGTTGTTCTTTTCTTCCGC	N/A	Sanger sequencing and DAS for *SdhC*	McCallum, Idnurm, and Van de Wouw (2025)
LmSdhC‐1F	ATCGAGACGTGACTAAGCAGC	N/A	Sanger sequencing and DAS for *SdhC*	McCallum, Idnurm, and Van de Wouw (2025)
LmsdhD‐1F	AAACCATTTTTCAGCGTTGTCG	N/A	Sanger sequencing and DAS for *SdhD*	McCallum, Idnurm, and Van de Wouw (2025)
LmsdhD‐1R	TATCAAGATGCCACGCCTAC	N/A	Sanger sequencing and DAS for *SdhD*	McCallum, Idnurm, and Van de Wouw (2025)
sdh_b_Fwd_2	TCATAACGTTCAGTAGGTACACCAGCTCGCCTCGTCTTAC	N/A	PCR *SdhB*	This study
sdh_b_Rev_2	TAAGATATCGAGCTCGGTACGGAACGTGGCTGTGGAGTTC	N/A	PCR *SdhB*	This study
sdh_d_Fwd_5	TCTAGAGGATCCCCGGGTACACAAGCTGCTCGTGGGGAATG	N/A	PCR *SdhD*	This study
sdh_d_Rev_3	TAAGATATCGAGCTCGGTACTATCAAGATGCCACGCCTAC	N/A	PCR *SdhD*	This study

Genomic DNA was extracted from each progeny using the DNeasy Plant extraction kit (Qiagen). Six bulked DNA samples were created by mixing progeny DNA in equal proportions and then sequenced at Australian Genome Research Facility (AGRF) using NovaSeq Control Software (NCS) v. 1.2.2.48004 and Real Time Analysis (RTA) v. 4.6.7. To identify QTLs associated with SDHI resistance, sequencing reads were trimmed, decontaminated and filtered (average quality > 35 and minimum length > 100) using *bbduk.sh* from BBMap v. 39.01 (Bushnell et al. 2017). The filtered reads were then mapped to the JN3 reference genome (Rouxel et al. 2011) using BWA (Li 2013). Variant discovery and SNP calling was carried out following the best practices of Genome Analysis ToolKit 4 (GATK4) (Van der Auwera et al. 2013; Van der Auwera and O'Connor 2020) with the bulks grouped into seven comparisons, each having a GATK4 generated .*vcf* output. The D2 × 24BL107 cross had a single comparison (S‐R) while the D3 × 24BL110 had six comparisons (SS‐IS, SS‐II, SS‐RR, RR‐IS, RR‐II and IS‐II). The .*vcf* files were converted to .*tsv* files retaining the necessary columns (CHROM, POS, REF, ALT, AD, DP, GQ) and analysed in R v. 4.4.2 (R Core Team 2024). The *QTLseqr* v. 0.7.5.2 (Mansfeld and Grumet 2018) package was used to identify significant QTLs using a G′ analysis.

### 4.6 Sanger Sequencing of *Sdh* Genes

The *SdhB, SdhC* and *SdhD* regions of 14 isolates (24BL105–24BL110, D2, D3, D4, D6, D13, D16, D17 and D20; Tables 2, S2) were sequenced. Genomic DNA of isolates was extracted using the DNeasy Plant Mini extraction kit (Qiagen) from mycelia. PCR primers (Table 4) designed to amplify the full coding region of each gene were used with the PCR conditions provided in Table S6. PCR product purification was performed using the QIAquick PCR Purification Kit (Qiagen). Amplicons were sequenced through Sanger chemistry by AGRF. Results were analysed in Geneious (v. R9.0.5).

### 4.7 KASP PCR Marker Development

KASP markers were developed for two SNPs in *SdhB* (*SdhB*‐H277Y and *SdhB*‐H277L) and one SNP in *SdhD* (*SdhD*‐D144G), using approaches as described by Van de Wouw et al. 2022. Primer assay mixes consisting of 12 μL of ‘primer_Res’ (100 nM), 12 μL of ‘primer_Sus’, 30 μL of ‘primer_com’ (100 nM) and 46 μL of 10 mM Tris–HCl (pH 8.3) were established for each resistance locus (Table 4). The KASP reactions consisted of 5 μL of 2× Master mix (KASP‐TF V4.0 Low ROX), 0.14 μL of Primer assay mix, 3.86 μL sterile water and 1.0 μL of DNA (1–2 ng μL^−1^). Three replicates of every sample were included in each assay run. All assays were carried out on either a Quantstudio 1 or Quantstudio 5 machine. All data were exported and analysed in Microsoft Excel (Tables S3–, S5).

### 4.8 Generation of Complemented Transformants

To validate the role of *SdhB* and *SdhD* mutations in conferring SDHI resistance, complemented transformants were generated. Five alleles (*SdhB*‐WT, *SdhB*‐H277Y, *SdhB*‐H277L, *SdhD‐*WT and *SdhD*‐D144G) were amplified by PCR using high‐fidelity Q5 polymerase (New England Biolabs) for three *SdhB* alleles and Ex Taq DNA polymerase (TaKaRa Bio) for two *SdhD* alleles. Genomic DNA of isolates D3, 24BL105, 24BL106 and 24BL110 were used as PCR templates. *SdhB*‐WT, *SdhB*‐H277Y and *SdhB*‐H277L were amplified from isolates D3, 24BL105 and 24BL106, respectively (Table 4), under PCR conditions in Table S7. *SdhD*‐WT and *SdhD*‐D144G were amplified from isolates D3 and 24BL110, respectively (Table 4), under PCR conditions in Table S8. Primers for PCR (Table 4) were designed in Geneious Prime v. 0.2025.0.3 from *SdhB* and *SdhD* reference sequences obtained from MycoCosm (Nordberg et al. 2014). PCR products were extracted with the QIAquick Gel Extraction Kit (Qiagen).

PCR amplicons were inserted into vectors linearised by KpnI, using the NEBuilder Hifi DNA Assembly kit (New England Biolabs) to generate complementation plasmids. The amplicons of *SdhB*‐WT, *SdhB*‐H277Y and *SdhB*‐H277L were inserted into plasmid pMAI6 containing a hygromycin resistance cassette. The amplicons of *SdhD*‐WT and *SdhD*‐D144G were inserted into plasmid pMAI2 containing a G418 resistance cassette (Idnurm et al. 2017). In total, five constructs were assembled for complementation. Assembled constructs were transformed into *Escherichia coli* cells (New England Biolabs) by the heat shock method following the NEBuilder HiFi DNA assembly chemical transformation protocol. Plasmid DNA from transformed *E. coli* was extracted using the QIAprep Spin Miniprep Kit protocol (Qiagen), and plasmids were further verified through whole plasmid sequencing (AGRF) as a small‐scale next‐generation sequencing (NGS) service. Alignments of NGS reads were inspected to confirm insertion of PCR products without errors.

To integrate complementation plasmids (pLmSdhB‐WT, pLmSdhB‐H277Y, pLmSdhB‐H277L, pLmSdhD‐WT and pLmSdhD‐D144G) into *L. maculans*, plasmids were transformed into *Agrobacterium tumefaciens* EHA105 using a chemical transformation process. Complementation plasmid DNA (1 μL) was mixed with 50 μL competent cells. Samples were left on ice for 5 min and transferred into liquid nitrogen for 5 min. Afterwards, tubes were incubated at 37°C for 5 min and then immediately added to 950 μL SOC Outgrowth Medium (New England Biolabs). The cultures were incubated at 28°C for 2 h shaking at 250 rpm then plated on selective Luria Bertani (LB) agar plates (LB, 2% agar, kanamycin) at 28°C for incubation for 2 days. *A. tumefaciens* colonies that grew on selective plates were picked up and screened by colony PCR (Table 4, Tables S7 and S8). *A. tumefaciens* ‐mediated transformation was conducted to generate complemented *L. maculans* transformants using modified methods (Gardiner and Howlett 2004). Six types of transformation were conducted: *SdhB*‐H277Y into D3, *SdhB*‐H277L into D3, *SdhD*‐D144G into D3, *SdhB*‐WT into 24BL105, *SdhB*‐WT into 24BL106 and *SdhD*‐WT into 79R103.

After 10–14 days, transformants were picked from the transformation plates. Eighteen complemented transformants were cultured on V8 agar plates with either hygromycin or G418, and cefotaxime. After 7 days, the spores from the plates were streaked onto new V8 selection plates then left for 7 days before single‐spore colonies were picked onto new V8 selection plates. To maintain complemented transformants, the isolates were cultured onto fresh V8 plates each week.

### 4.9 Detection of Gene Expression in Transformants

All transformants were screened by reverse transcription‐quantitative PCR (RT‐qPCR) and KASP PCR to ensure the expression of both the resistant and susceptible alleles in complemented isolates. Transformants and parent isolates (D3, 24BL105, 24BL106 and 79R103) were cultured in 10% cleared liquid V8 medium for a week before mycelium was freeze‐dried. RNA was extracted from samples using the RNeasy Plant Mini Kit (Qiagen). After RNA extraction, samples were treated with DNase I (Thermo Fisher Scientific) to remove DNA contamination and cDNA was synthesised with SuperScript IV reverse transcriptase (Thermo Fisher Scientific). The qPCR was performed using the SYBR FAST Universal qPCR kit (KAPA Biosystems) on a CFX95 Real‐Time System PCR machine (Bio‐Rad) with qPCR primers (Table 4) and PCR conditions (Figures S2 and S3). The developed KASP markers (Table 4) were also used to detect the gene expression in transformant and control cDNA using primers and conditions as above. Whilst the specific gene expression levels could not be determined, if both alleles are expressed then the KASP markers would indicate a heterozygote, that is, both alleles detected.

### 4.10 Calibration Populations

Previously developed markers were used for identification and quantification of mutations in *Sdh* gene regions (McCallum, Idnurm, and Van de Wouw 2025). Mock calibration populations were developed to test the limits for accurate detection of SDHI resistance alleles. Populations containing mixtures of 24BL110 (*SdhB*‐H277L and *SdhD*‐D144G) DNA and D2 (wild type) DNA were created through a serial dilution resulting in seven samples with the frequency of 24BL110 DNA varying from 100% to 0.0001% (10‐fold dilution, Table S9). The genomic DNA mixtures were sequenced at AGRF, generating 150 bp reads using Illumina sequencing, and the reads were analysed as previously described (McCallum, Idnurm, and Van de Wouw 2025).

### 4.11 DAS Markers for Population Monitoring

Ascospores were liberated from the 110 canola stubble samples from the 2024 Australian national fungicide resistance population survey using a Burkard spore liberator, described in McCredden et al. (2018). Nine stubble samples failed to release enough ascospores, leaving 101 ascospore populations. After liberation, ascospores were germinated in V8 liquid medium (10% V8 juice) for 48 h and then the mycelium was freeze‐dried. Genomic DNA was extracted from the freeze‐dried mycelium using the DNeasy Plant Mini extraction kit (Qiagen). *SdhB* and *SdhD* genes were amplified using primers in Table 4 and analysed by DAS following the protocol in McCallum, Idnurm, and Van de Wouw (2025). Gene regions were amplified and purified as described in Section 4.6. Amplicons were sequenced using a NovaSeq X lane (AGRF, Australia), generating 150 bp paired‐end reads. Reads were analysed in a similar pipeline as previously described (McCallum, Idnurm, and Van de Wouw 2025; Scanlan et al. 2023). A list of all known genotypes (Data S1) was used as an index for quantification of allele frequencies in each ascospore population. Statistical analysis was completed in R v. 4.4.2 (R Core Team 2024) using the *tidyverse* package (Wickham et al. 2019). Correlations between RS scores from the fungicide resistance survey and frequency of SDHI mutants in the populations were determined using the *cor.test* function in the *stats* package (R Core Team 2024), using the ‘spearman’ method.

## Author Contributions

**Ko‐Hsuan Tsao:** conceptualisation, methodology, investigation, validation, formal analysis, funding acquisition, visualisation, writing – original draft, writing – review and editing. **Alexander Idnurm:** conceptualisation, investigation, supervision, funding acquisition, project administration, writing – review and editing. **Angela P. Van de Wouw:** conceptualisation, data curation, investigation, validation, formal analysis, supervision, funding acquisition, project administration, writing – review and editing. **Alec J. McCallum:** conceptualisation, methodology, data curation, investigation, validation, formal analysis, funding acquisition, visualisation, writing – original draft, writing – review and editing.

## Funding

We thank the Australian Research Council (IM230100025), industry partners Syngenta and Marcroft Grains Pathology, the Grains Research and Development Corporation (GRDC) (MGP2504‐0022RTX) and the GRDC Research Scholarships (UOM2304‐004RSX and UOM2601‐007RSX) for funding.

## Conflicts of Interest

The authors declare no conflicts of interest.

## Supporting information


**Data S1:** A list of all known genotypes used as an index for quantification of allele frequencies in each ascospore population.

**Figure S1:** Kompetitive Allele Specific PCR (KASP) results for the progeny of the D2 × 24BL107 cross (A) and the D3 × 24BL110 cross (B, C). Progeny are classed as mutant (H277Y in A, H277L in B, D144G in C; solid square) or wild‐type (WT, solid triangle) with parents indicated as control (hollow square) or WT control (hollow triangle). No template controls (NTC, hollow diamond) are also included.

**Figure S2:** Change in *SdhB* expression before and after SDHI treatment in transformants. Transformants were grouped according to the mutant allele present: *SdhB*‐H277Y, *SdhB*‐H277L and *SdhD*‐D144G. The quantitative PCR conditions were 95°C for 30 s; then 40 cycles of 95°C for 15 s, 60°C for 30 s; then 65°C to 95°C for 5 s. Changes of expression were calculated as (−Δ*C*_*t*_ of SDHI‐treated) minus (−Δ*C*_*t*_ of non‐treated). Δ*C*_*t*_ was calculated as the *C*_*t*_ value of *SdhB* minus the *C*_*t*_ value of *Actin* (reference gene).

**Figure S3:** Change in *SdhD* expression before and after SDHI treatment in transformants. Transformants were grouped according to the mutant allele present: *SdhB*‐H277Y, *SdhB*‐H277L and *SdhD*‐D144G. The quantitative PCR conditions were 95°C for 30 s; then 40 cycles of 95°C for 15 s, 60°C for 30 s; then 65°C to 95°C for 5 s. Changes of expression were calculated as (−Δ*C*_*t*_ of SDHI‐treated) minus (−Δ*C*_*t*_ of non‐treated). Δ*C*_*t*_ was calculated as the *C*_*t*_ value of *SdhD* minus the *C*_*t*_ value of *Actin* (reference gene).

**Figure S4:** Representative visual examples of the in vitro sensitivity assays for D3 (susceptible) and 24BL110 (resistant) isolate exposed to fluopyram (0–1280 ng mL^−1^) and pydiflumetofen (0–150 ng mL^−1^).

**Figure S5:** Calibration samples for deep amplicon sequencing showing the frequency of the resistance alleles in 24BL110 isolate. The *SdhB* (orange circle) allele is *SdhB*‐H277L and the *SdhD* (blue triangle) allele is *SdhD*‐D144G. The solid black line represents a 1:1 ratio. Each sample was sequenced and both alleles quantified with the expected allele frequency compared to the observed allele frequency. The estimation of allele frequency is accurate between 0.1% and 100% resistance frequency but the observed frequency plateaus between 0.1% and 0.01%. Mutations that are not present in the D2 or 24BL110 can be detected at frequencies up to a maximum of 0.1%, likely due to PCR errors, sequencing errors or index hopping. DAS assay can accurately identify mutations that are present at frequencies of at least 0.1%.

**Table S1:** Details of the QTLs from both crosses and the seven total comparisons.

**Table S2:** Details of susceptible isolates.

**Table S3:** Detection of *SdhB*‐H277Y in complemented transformants by KASP markers. The run condition was 1 cycle at 94°C for 15 min (denaturing), 10 cycles of 94°C for 20 s; 61°C–55°C (decreasing 0.6°C each cycle) for 60 s (PCR cycles), 26 cycles of 94°C for 20 s; 55°C for 60 s (PCR cycles), 1 cycle of 25°C for 60 s (cooling), 1 cycle of 25°C for 30 s (data acquiring).

**Table S4:** Detection of *SdhB*‐H277L in complemented transformants by KASP markers. The run condition was 1 cycle at 94°C for 15 min (denaturing), 10 cycles of 94°C for 20 s; 61°C–55°C (decreasing 0.6°C each cycle) for 60 s (PCR cycles), 26 cycles of 94°C for 20 s; 55°C for 60 s (PCR cycles), 1 cycle of 25°C for 60 s (cooling), 1 cycle of 25°C for 30 s (data acquiring).

**Table S5:** Detection of *SdhD*‐D144G complemented transformants by KASP markers. The run condition was 1 cycle at 94°C for 15 min (denaturing), 10 cycles of 94°C for 20 s; 61°C–55°C (decreasing 0.6°C each cycle) for 60 s (PCR cycles), 26 cycles of 94°C for 20 s; 55°C for 60 s (PCR cycles), 1 cycle of 25°C for 60 s (cooling), 1 cycle of 25°C for 30 s (data acquiring).

**Table S6:** PCR conditions for *Sdh* genes for Sanger sequencing.

**Table S7:** PCR conditions for *SdhB*‐WT/H277Y/H277L amplification.

**Table S8:** PCR conditions for *SdhD*‐WT/D144G amplification.

**Table S9:** Details of the frequency of 24BL110 and D2 in calibration populations.

## Data Availability

The data that support the findings of this study are available from the corresponding author upon reasonable request.
