# Supplementary material for: Mutations in the SdhB and SdhD Genes Confer Resistance to Succinate Dehydrogenase Inhibitor Fungicides in Australian Populations of Leptosphaeria maculans
Source: Mol Plant Pathol. 2026 Nov 6;27(11):e70364. doi: 10.1111/mpp.70364 (PMC13743630; doi:10.1111/mpp.70364)
Supplement: Supplementary file 2 [file MPP-27-e70364-s002.docx]

**Table S9:** Details of the frequency of 24BL110 and D2 in calibration populations.

| Sample No. | Frequency of 24BL110 (%) | Frequency of D2 (%) |
| --- | --- | --- |
| 1 | 100 | 0 |
| 2 | 10 | 90 |
| 3 | 1 | 99 |
| 4 | 0.1 | 99.9 |
| 5 | 0.01 | 99.99 |
| 6 | 0.001 | 99.999 |
| 7 | 0.0001 | 99.9999 |
