# Supplementary material for: Mutations in the SdhB and SdhD Genes Confer Resistance to Succinate Dehydrogenase Inhibitor Fungicides in Australian Populations of Leptosphaeria maculans
Source: Mol Plant Pathol. 2026 Nov 6;27(11):e70364. doi: 10.1111/mpp.70364 (PMC13743630; doi:10.1111/mpp.70364)
Supplement: Supplementary file 3 [file MPP-27-e70364-s005.docx]

**Table S1:** Details of the QTLs from both crosses and the seven total comparisons.

| **Cross** | **Bulks** | **QTL contig** | **QTL start** | **QTL length** | **n SNPs** |
| --- | --- | --- | --- | --- | --- |
| D2 × 24BL107 | R + S | SC00 | 1304546 | 326382 | 897 |
| D3 × 24BL110 | SS + RR | SC00 | 1349152 | 281776 | 205 |
|  |  | SC03 | 1666942 | 320945 | 137 |
|  | SS + II | SC00 | 1284340 | 346588 | 147 |
|  |  |  | 1710164 | 360971 | 375 |
|  | SS + IS | SC03 | 1681171 | 116477 | 54 |
|  |  |  | 1934099 | 33602 | 15 |
|  |  | SC07 | 1577900 | 29200 | 10 |
|  | RR + II | SC03 | 1680755 | 63996 | 23 |
|  |  |  | 1809889 | 124210 | 59 |
|  | RR + IS | SC00 | 1488677 | 122464 | 189 |
|  | IS + II | SC00 | 1496917 | 130423 | 160 |
|  |  |  | 1889365 | 18784 | 7 |
|  |  | SC03 | 2423572 | 211402 | 222 |
|  |  | SC07 | 907427 | 10878 | 6 |
