# Supplementary material for: Mutations in the SdhB and SdhD Genes Confer Resistance to Succinate Dehydrogenase Inhibitor Fungicides in Australian Populations of Leptosphaeria maculans
Source: Mol Plant Pathol. 2026 Nov 6;27(11):e70364. doi: 10.1111/mpp.70364 (PMC13743630; doi:10.1111/mpp.70364)
Supplement: Supplementary file 5 [file MPP-27-e70364-s009.docx]

**Table S8:** PCR conditions for *SdhD*-WT/D144G amplification.

| Steps | Time | Temperature (°C) | Cycle |
| --- | --- | --- | --- |
| Initial denaturation | 30 seconds | 98 | 1 |
| Denaturation | 10 seconds | 98 | 30 |
| Annealing | 30 seconds | 60 |  |
| Extension | 3 minutes | 72 |  |
| Final incubation | 2 minutes | 72 | 1 |
