# Supplementary material for: Mutations in the SdhB and SdhD Genes Confer Resistance to Succinate Dehydrogenase Inhibitor Fungicides in Australian Populations of Leptosphaeria maculans
Source: Mol Plant Pathol. 2026 Nov 6;27(11):e70364. doi: 10.1111/mpp.70364 (PMC13743630; doi:10.1111/mpp.70364)
Supplement: Supplementary file 6 [file MPP-27-e70364-s011.docx]

**Table S5**: Detection of *SdhD*-D144G complemented transformants by KASP markers. The run condition was 1 cycle at 94°C for 15 minutes (denaturing), 10 cycles of 94°C for 20 sec; 61-55°C (decreasing 0.6°C each cycle) for 60 sec (PCR cycles), 26 cycles of 94°C for 20 sec; 55°C for 60 sec (PCR cycles), 1 cycle of 25°C for 1 minute (cooling), 1 cycle of 25°C for 30 second (data acquiring).

| Sample | Expected genotype | Wild-type allele delta run | *SdhD*-D144G delta run | KASP detected genotype |
| --- | --- | --- | --- | --- |
| D3 gDNA | *SdhD*-WT | 3.032 | 0.244 | *SdhD*-WT |
| 79R103 cDNA | *SdhD*-D144G | 0.346 | 5.335 | *SdhD*-D144G |
| D3+*SdhD*-D144G_1 cDNA | *SdhD*-WT + *SdhD*-D144G | 0.672 | 4.239 | *SdhD*-WT + *SdhD*-D144G |
| D3+*SdhD*-D144G_2 cDNA | *SdhD*-WT + *SdhD*-D144G | 0.789 | 2.965 | *SdhD*-WT + *SdhD*-D144G |
| D3+*SdhD*-D144G_3 cDNA | *SdhD*-WT + *SdhD*-D144G | 1.012 | 3.973 | *SdhD*-WT + *SdhD*-D144G |
| 79R103+*SdhD*-WT_1 cDNA | *SdhD*-D144G +*SdhD*-WT | 0.926 | 2.902 | *SdhD*-D144G +*SdhD*-WT |
| 79R103+*SdhD*-WT_2 cDNA | *SdhD*-D144G +*SdhD*-WT | 0.870 | 2.746 | *SdhD*-D144G +*SdhD*-WT |
| 79R103+*SdhD*-WT_3 cDNA | *SdhD*-D144G +*SdhD*-WT | 0.879 | 3.238 | *SdhD*-D144G +*SdhD*-WT |
| No template control (NTC) | N/A | 0321 | 0.295 | N/A |
