# Supplementary material for: Mutations in the SdhB and SdhD Genes Confer Resistance to Succinate Dehydrogenase Inhibitor Fungicides in Australian Populations of Leptosphaeria maculans
Source: Mol Plant Pathol. 2026 Nov 6;27(11):e70364. doi: 10.1111/mpp.70364 (PMC13743630; doi:10.1111/mpp.70364)
Supplement: Supplementary file 7 [file MPP-27-e70364-s012.docx]

**Table S2:** Details of susceptible isolates.

| Isolate name | Synonyms | Year collected | Location |
| --- | --- | --- | --- |
| D2 | IBCN15 | 1988 | Streatham, Victoria |
| D3 | IBCN16 | 1988 | Mt Barker, Western Australia |
| D4 | IBCN17 | 1988 | Millicent, South Australia |
| D6 | IBCN75 | 1987 | Mt Barker, Western Australia |
| D13 | 09SMW024 | 2009 | Cummins, South Australia |
| D16 | 13W016 | 2013 | Wangary, South Australia |
| D17 | 13P073 | 2013 | Wangary, South Australia |
| D20 | 53R07 | - | Generated from *in vitro* cross |
