# Supplementary material for: Mutations in the SdhB and SdhD Genes Confer Resistance to Succinate Dehydrogenase Inhibitor Fungicides in Australian Populations of Leptosphaeria maculans
Source: Mol Plant Pathol. 2026 Nov 6;27(11):e70364. doi: 10.1111/mpp.70364 (PMC13743630; doi:10.1111/mpp.70364)
Supplement: Supplementary file 8 [file MPP-27-e70364-s014.docx]

**Table S3:** Detection of *SdhB*-H277Y in complemented transformants by KASP markers. The run condition was 1 cycle at 94°C for 15 minutes (denaturing), 10 cycles of 94°C for 20 sec; 61-55°C (decreasing 0.6°C each cycle) for 60 sec (PCR cycles), 26 cycles of 94°C for 20 sec; 55°C for 60 sec (PCR cycles), 1 cycle of 25°C for 1 minute (cooling), 1 cycle of 25°C for 30 second (data acquiring).

| Sample | Expected genotype | Wild-type allele amplification | *SdhB*-H277Y allele amplification | KASP detected genotype |
| --- | --- | --- | --- | --- |
| D3 gDNA | *SdhB*-WT | 3.104 | 0.716 | *SdhB*-WT |
| 24BL105 gDNA | *SdhB*-H277Y | 0.507 | 6.952 | *SdhB*-H277Y |
| 24BL105 cDNA | *SdhB*-H277Y | 0.402 | 4.914 | *SdhB*-H277Y |
| 79R103 cDNA | *SdhD*-D144G | 2.911 | 0.850 | *SdhB*-WT |
| D3+*SdhB*-H277Y_1 cDNA | *SdhB*-WT + *SdhB*-H277Y | 1.909 | 2.868 | *SdhB*-WT + *SdhB*-H277Y |
| D3+*SdhB*-H277Y_2 cDNA | *SdhB*-WT + *SdhB*-H277Y | 1.940 | 3.249 | *SdhB*-WT + *SdhB*-H277Y |
| D3+*SdhB*-H277Y_3 cDNA | *SdhB*-WT + *SdhB*-H277Y | 2.023 | 3.060 | *SdhB*-WT + *SdhB*-H277Y |
| 24BL105+*SdhB*-WT_1 cDNA | *SdhB*-H277Y + *SdhB*-WT | 1.904 | 1.814 | *SdhB*-WT + *SdhB*-H277Y |
| 24BL105+*SdhB*-WT_2 cDNA | *SdhB*-H277Y + *SdhB*-WT | 2.163 | 2.515 | *SdhB*-WT + *SdhB*-H277Y |
| 24BL105+*SdhB*-WT_3 cDNA | *SdhB*-H277Y + *SdhB*-WT | 2.102 | 3.647 | *SdhB*-WT + *SdhB*-H277Y |
| No template control (NTC) | N/A | 0.285 | 0.510 | NTC |
