# Supplementary material for: Mutations in the SdhB and SdhD Genes Confer Resistance to Succinate Dehydrogenase Inhibitor Fungicides in Australian Populations of Leptosphaeria maculans
Source: Mol Plant Pathol. 2026 Nov 6;27(11):e70364. doi: 10.1111/mpp.70364 (PMC13743630; doi:10.1111/mpp.70364)
Supplement: Supplementary file 9 [file MPP-27-e70364-s015.docx]

**Table S4:** Detection of *SdhB*-H277L in complemented transformants by KASP markers. The run condition was 1 cycle at 94°C for 15 minutes (denaturing), 10 cycles of 94°C for 20 sec; 61-55°C (decreasing 0.6°C each cycle) for 60 sec (PCR cycles), 26 cycles of 94°C for 20 sec; 55°C for 60 sec (PCR cycles), 1 cycle of 25°C for 1 minute (cooling), 1 cycle of 25°C for 30 second (data acquiring).

| Sample | Expected genotype | Wild-type allele delta run | *SdhB*-H277L delta run | KASP detected genotype |
| --- | --- | --- | --- | --- |
| D3 gDNA | *SdhB*-WT | 2.713 | 0.347 | *SdhB*-WT |
| 24BL105 gDNA | *SdhB*-H277Y | 2.386 | 0.278 | *SdhB*-H277Y |
| 24BL106 cDNA | *SdhB*-H277L | 0.397 | 2.152 | *SdhB*-H277L |
| D3+*SdhB*-H277L_1 cDNA | *SdhB-*WT + *SdhB*-H277L | 0.774 | 3.255 | *SdhB*-WT +*SdhB*-H277L |
| D3+*SdhB*-H277L_2cDNA | *SdhB*-WT + *SdhB*-H277L | 0.819 | 1.702 | *SdhB*-WT + *SdhB*-H277L |
| D3+*SdhB*-H277L_3cDNA | *SdhB*-WT + *SdhB*-H277L | 0.917 | 4.220 | *SdhB*-WT +*SdhB*-H277L |
| 24BL106+*SdhB*-WT_1 cDNA | *SdhB*-H277L + *SdhB*-WT | 1.334 | 2.154 | *SdhB*-H277L+ *SdhB*-WT |
| 24BL106+*SdhB*-WT_2 cDNA | *SdhB*-H277L + *SdhB*-WT | 1.494 | 2.183 | *SdhB*-H277L+ *SdhB*-WT |
| 24BL106+*SdhB*-WT_3 cDNA | *SdhB*-H277L + *SdhB*-WT | 2.129 | 1.701 | *SdhB*-H277L+ *SdhB*-WT |
| No template control (NTC) | N/A | 0.371 | 0.477 | NTC |
